# A fast least-squares algorithm for population inference

**DOI:** 10.1186/1471-2105-14-28

**Published:** 2013-01-23

**Authors:** R Mitchell Parry, May D Wang

**Affiliations:** 1 The Wallace H. Coulter Department of Biomedical Engineering, Georgia Institute of Technology and Emory University, Atlanta, GA 30332, USA; 2 Parker H. Petit Institute of Bioengineering and Biosciences and Department of Electrical and Computer Engineering, Georgia Institute of Technology, Atlanta, GA 30332, USA; 3 Winship Cancer Institute and Hematology and Oncology Department, Emory University, 30322, Atlanta, GA, USA

## Abstract

**Background:**

Population inference is an important problem in genetics used to remove population stratification in genome-wide association studies and to detect migration patterns or shared ancestry. An individual’s genotype can be modeled as a probabilistic function of ancestral population memberships, **Q**, and the allele frequencies in those populations, **P**. The parameters, **P** and **Q**, of this binomial likelihood model can be inferred using slow sampling methods such as Markov Chain Monte Carlo methods or faster gradient based approaches such as sequential quadratic programming. This paper proposes a least-squares simplification of the binomial likelihood model motivated by a Euclidean interpretation of the genotype feature space. This results in a faster algorithm that easily incorporates the degree of admixture within the sample of individuals and improves estimates without requiring trial-and-error tuning.

**Results:**

We show that the expected value of the least-squares solution across all possible genotype datasets is equal to the true solution when part of the problem has been solved, and that the variance of the solution approaches zero as its size increases. The Least-squares algorithm performs nearly as well as *Admixture* for these theoretical scenarios. We compare least-squares, *Admixture*, and *FRAPPE* for a variety of problem sizes and difficulties. For particularly hard problems with a large number of populations, small number of samples, or greater degree of admixture, least-squares performs better than the other methods. On simulated mixtures of real population allele frequencies from the HapMap project, *Admixture* estimates sparsely mixed individuals better than Least-squares. The least-squares approach, however, performs within 1.5% of the *Admixture* error. On individual genotypes from the HapMap project, *Admixture* and least-squares perform qualitatively similarly and within 1.2% of each other. Significantly, the least-squares approach nearly always converges 1.5- to 6-times faster.

**Conclusions:**

The computational advantage of the least-squares approach along with its good estimation performance warrants further research, especially for very large datasets. As problem sizes increase, the difference in estimation performance between all algorithms decreases. In addition, when prior information is known, the least-squares approach easily incorporates the expected degree of admixture to improve the estimate.

## Background

The inference of population structure from the genotypes of admixed individuals poses a significant problem in population genetics. For example, genome wide association studies (GWAS) compare the genetic makeup of different individuals in order to extract differences in the genome that may contribute to the development or suppression of disease. Of particular interest are single nucleotide polymorphisms (SNPs) that reveal genetic changes at a single nucleotide in the DNA chain. When a particular SNP variant is associated with a disease, this may indicate that the gene plays a role in the disease pathway, or that the gene was simply inherited from a population that is more (or less) predisposed to the disease. Determining the inherent population structure within a sample removes confounding factors before further analysis and reveals migration patterns and ancestry [1]. This paper deals with the problem of inferring the proportion of an individual’s genome originating from multiple ancestral populations and the allele frequencies in these ancestral populations from genotype data.

Methods for revealing population structure are divided into fast multivariate analysis techniques and slower discrete admixture models [2]. Fast multivariate techniques such as principal components analysis (PCA) [2-8] reveal subspaces in the genome where large differences between individuals are observed. For case–control studies, the largest differences commonly due to ancestry are removed to reduce false positives [4]. Although PCA provides a fast solution, it does not directly infer the variables of interest: the population allele frequencies and individual admixture proportions. On the other hand, discrete admixture models that estimate these variables typically require much more computation time. Following a recent trend toward faster gradient-based methods, we propose a faster simpler least-squares algorithm for estimating both the population allele frequencies and individual admixture proportions.

Pritchard et al. [9] originally propose a discrete admixture likelihood model based on the random union of gametes for the purpose of population inference. In particular, their model assumes Hardy-Weinberg equilibrium within the ancestral populations (*i.e.,* allele frequencies are constant) and linkage equilibrium between markers within each population (*i.e.,* markers are independent). Each individual in the current sample is modeled as having some fraction of their genome originating from each of the ancestral populations. The goal of population inference is to estimate the ancestral population allele frequencies, **P**, and the admixture of each individual, **Q**, from the observed genotypes, **G**. If the population of origin for every allele, **Z**, is known, then the population allele frequencies and the admixture for each individual have a Dirichlet distribution. If, on the other hand, **P** and **Q** are known, the population of origin for each individual allele has a multinomial distribution. Pritchard et al. infer populations by alternately sampling **Z** from a multinomial distribution based on **P** and **Q**; and **P** and **Q** from Dirichlet distributions based on **Z**. Ideally, this Markov Chain Monte Carlo sampling method produces independent identically distributed samples (**P**,**Q**) from the posterior distribution *P*(**P**,**Q**|**G**). The inferred parameters are taken as the mean of the posterior. This algorithm is implemented in an open-source software tool called *Structure*[9].

The binomial likelihood model proposed by Pritchard et al. was originally used for datasets of tens or hundreds of loci. However, as datasets become larger, especially considering genome-wide association studies with thousands or millions of loci, two problems emerge. For one, linkage disequilibrium introduces correlations between markers. Although Falush et al. [10] extended *Structure* to incorporate loose linkage between loci, larger datasets also pose a computational challenge that has not been met by these sampling-based approaches. This has led to a series of more efficient optimization algorithms for the same likelihood model with uncorrelated loci. This paper focuses on improving computational performance, leaving the treatment of correlated loci to future research.

Tang et al. [11] proposed a more efficient expectation maximization (EM) approach. Instead of randomly sampling from the posterior distribution, the *FRAPPE* EM algorithm [11] starts with a randomly initialized **Z**, then alternates between updating the values of **P** and **Q** for fixed **Z**, and maximizing the likelihood of **Z** for fixed **P** and **Q**. Their approach achieves similar accuracy to *Structure* and requires much less computation time. Wu et al. [12] specialized the EM algorithm in *FRAPPE* to accommodate the model without admixture, and generalized it to have different mixing proportions at each locus. However, these EM algorithms estimate an unnecessary and unobservable variable **Z**, something that more efficient algorithms could avoid.

Alexander et al. [13] proposed an even faster approach for inferring **P** and **Q** using the same binomial likelihood model but bypassing the unobservable variable **Z**. Their close-source software, *Admixture*, starts at a random feasible solution for **P** and **Q** and then alternates between maximizing the likelihood function with respect to **P** and then maximizing it with respect to **Q**. The likelihood is guaranteed not to decrease at each step eventually converging at a local maximum or saddle point. For a moderate problem of approximately 10000 loci, *Admixture* achieves comparable accuracy to *Structure* and requires only minutes to execute compared to hours for *Structure*[13].

Another feature of *Structure*’s binomial likelihood model is that it allowed the user to input prior knowledge about the degree of admixture. The prior distribution for **Q** takes the form of a Dirichlet distribution with a degree of admixture parameter, *α*, for every population. For *α* = 0, all of an individual’s alleles originate from the same ancestral population; for *α* > 0, individuals contain a mixture of alleles from different populations; for *α* = 1, every assignment of alleles to populations is equally likely (i.e., the non-informative prior); and for *α* → ∞, all individuals have equal contributions from every ancestral population. Alexander et al. replace the population degree of admixture parameter in *Structure* with two parameters, *λ* and *γ*, that when increased also decrease the level of admixture of the resulting individuals. However, the authors admit that tuning these parameters is non-trivial [14].

This paper contributes to population inference research by (1) proposing a novel least-squares simplification of the binomial likelihood model that results in a faster algorithm, and (2) directly incorporating the prior parameter *α* that improves estimates without requiring trial-and-error tuning. Specifically, we utilize a two block coordinate descent method [15] to alternately minimize the criterion for **P** and then for **Q**. We adapt a fast non-negative least-squares algorithm [16] to additionally include a sum-to-one constraint for **Q** and an upper-bound for **P**. We show that the expected value for the estimates of **P** (or **Q)** across all possible genotype datasets are equal to the true values when **Q** (or **P**) are known and that the variance of this estimate approaches zero as the problem size increases. Compared to *Admixture*, the least-squares approach provides a slightly worse estimate of **P** or **Q** when the other is known. However, when estimating **P** and **Q** from only the genotype data, the least-squares approach sometimes provides better estimates, particularly with a large number of populations, small number of samples, or more admixed individuals. The least-squares approximation provides a simpler and faster algorithm, and we provide it as Matlab scripts on our website.

## Results

First, we motivate a least-squares simplification of the binomial likelihood model by deriving the expected value and covariance of the least-squares estimate across all possible genotype matrices for partially solved problems. Second, we compare least-squares to sequential quadratic programming (*Admixture*’s optimization algorithm) for these cases. Third, we compare *Admixture*, *FRAPPE*, and least-squares using simulated datasets with a factorial design varying dataset properties in **G**. Fourth, we compare *Admixture* and least-squares using real population allele frequencies from the HapMap Phase 3 project. Finally, we compare the results of applying *Admixture* and least-squares to real data from the HapMap Phase 3 project where the true population structure is unknown.

The algorithms we discuss accept as input the number of populations, *K*, and the genotypes, *g*_*li*_∈{0,1,2}, representing the number of copies of the reference allele at locus *l* for individual *i*. Then, the algorithms attempt to infer the population allele frequencies, *p*_*lk*_ = [0,1], for locus *l* and population *k*, as well as the individual admixture proportions, *q*_*ki*_ = [0,1] where ∑_*k*_*q*_*ki*_ = 1. In all cases, 1 ≤ *l* ≤ *M*, 1 ≤ *i* ≤ *N*, and 1 ≤ *k* ≤ *K.* Table 1 summarizes the matrix notation.

**Table 1:** Matrix notation

**Genotype matrix**	**Population allele frequencies matrix**	**Individual admixture matrix**
$$G=\left[\begin{array}{l} g_{11}\;g_{12}\;\cdots \;g_{1N}\\ g_{21}\;g_{22}\;\cdots \;g_{2N}\\ \;\vdots \;\vdots \;\ddots \;\vdots \\ g_{M1}\;g_{M2}\;\cdots \;g_{\text{MN}} \end{array}\;\right]$$	$$P=\left[\begin{array}{l} p_{11}\;p_{12}\;\cdots \;p_{1K}\\ p_{21}\;p_{22}\;\cdots \;p_{2K}\\ \;\vdots \;\vdots \;\ddots \;\vdots \\ p_{M1}\;p_{M2}\;\cdots \;p_{\text{MK}} \end{array}\;\right]$$	$$Q=\left[\begin{array}{l} q_{11}\;q_{12}\;\cdots \;q_{1N}\\ q_{21}\;q_{22}\;\cdots \;q_{2N}\\ \;\vdots \;\vdots \;\ddots \;\vdots \\ q_{M1}\;q_{M2}\;\cdots \;q_{\text{KN}} \end{array}\;\right]$$
*g*_*li*_ ∈ {0, 1, 2} : number of reference alleles at *l*th locus for *i*th individual.	$0\leq P_{\mathrm{lk}}\leq 1$ : percentage of reference alleles at *l*th locus in *k*th population.	$q_{\text{ki}}\geq 0,\overset{K}{\underset{k=1}{\Sigma }}q_{\text{ki}}=1$ : fraction of *i*th individual’s genome originating from *k*th population.
*M* = number of loci (markers)		1≤l≤M
*N* = number of individuals		1≤i≤N
*K* = number of populations		1≤k≤K

### Empirical estimate and upper bound on total variance

To validate our derived bounds on the total variance (Equations 13, 17, 18 and 19), we generate simulated genotypes from a known target for **p** = [0.1, 0.7]^*T*^. We simulate *N* individual genotypes using the full matrix **Q** with each column drawn from a Dirichlet distribution with shape parameter *α*. We repeat the experiment 10000 times producing an independent and identically distributed genotype each time. Each trial produces one estimate for **p**. We then compute the mean and covariance of the estimates of **p** and compare them to those predicted in the bounds. For *α* = 1 and *N* = 100,

(1) $$\begin{array}{l} \text{mean}(\hat{p})=\left[\begin{array}{l} 0.0999\\ 0.7002 \end{array}\right]\\ \text{cov}(\hat{p})=\left[\begin{array}{l} 0.0027\;-0.0015\\ -0.0015\;0.0046 \end{array}\;\right]\\ \text{trace}(\text{cov}\left[\hat{p}\right])=0.0073 \end{array}$$

The bound using the sample covariance of **q** in Equation 13 provides the following:

(2) $$\begin{array}{l} QQ^{T}=\left[\begin{array}{l} 36.62\;16.20\\ 16.20\;30.99 \end{array},\;\right]\\ \text{trace}(\text{cov}\left[\hat{p}\right])\leq 0.0097 \end{array}$$

The bound using the properties of the Dirichlet distribution in Equation 17 provides a bound of 0.01. As the number of samples increases, the difference between the bound and the asymptotic bound for the Dirichlet distributed **q** will approach zero.

**Figure 1:**
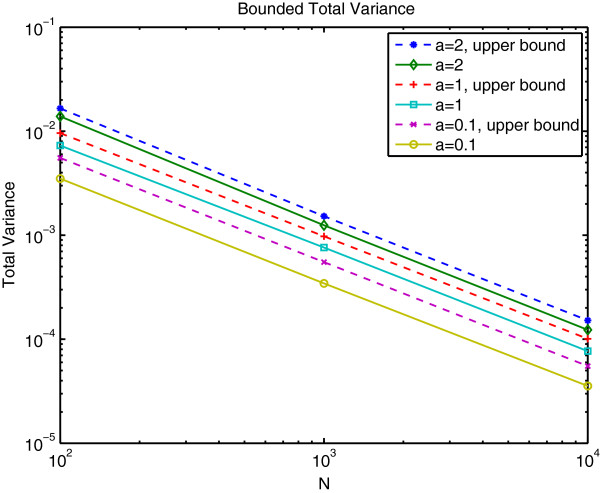
**Bound on total variance. **Solid and dashed lines correspond to the empirical estimate of the total variance and the upper bound for total variance, respectively.

Figure 1 plots the total variance (trace of the covariance) matrix for a variety of values for *N* and *α* using the same target value for **p**. Because the expected value of the estimate is equal to the true value of **p**, the total variance is analogous to the sum of the squared error (SSE) between the true **p** and its estimate. Clearly, the total variance decreases with *N*. For *N* = 10000, the root mean squared error falls below 1%.

Intuitively, the error in the least-squares estimate for **P** and **Q** decreases as the number of individuals and the number of loci increases, respectively. Figure 1 supports this notion, suggesting that on very large problems for which the gradient based and expectation maximization algorithms were designed, the error in the least-squares estimate approaches zero.

### Comparing least-squares approximation to binomial likelihood model

Given estimates of the population allele frequencies, early research focused on estimating the individual admixture [17]. We also note that the number of iterations and convergence properties confound the comparison of iterative algorithms. To avoid these problems and emulate a practical research scenario, we compare least-squares to sequential quadratic programming (used in *Admixture*) when **P** or **Q** are known *a priori.* In this scenario, each algorithm converges in exactly one step making it possible to compare the underlying updates for **P** and **Q** independently. For *N* = 100, 1000, and 10000; and *α* = 0.1, 1, and 2; we consider a grid of two-dimensional points for **p**, where *p*_*i*_ = {0.05, 0.15, …, 0.95}. For each trial, we first generate a random **Q** such that every column is drawn from a Dirichlet distribution with shape parameter, *α*. Then, we randomly generate a genotype using Equation 11. We compute the least-squares solution using Equation 27 and use Matlab’s built-in function ‘fmincon’ to minimize the negative of the log-likelihood in Equation 7, similar to *Admixture*’s approach. We repeat the process for 1000 trials and aggregate the results.

**Figure 2:**
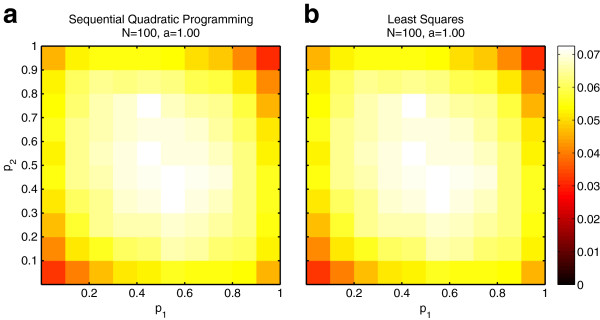
**Precision of best-case scenario for estimating P. **Root mean squared error for different values of p using (**a**) *Admixture*’s Sequential Quadratic Programming or (**b**) the least-squares approximation.

**Figure 3:**
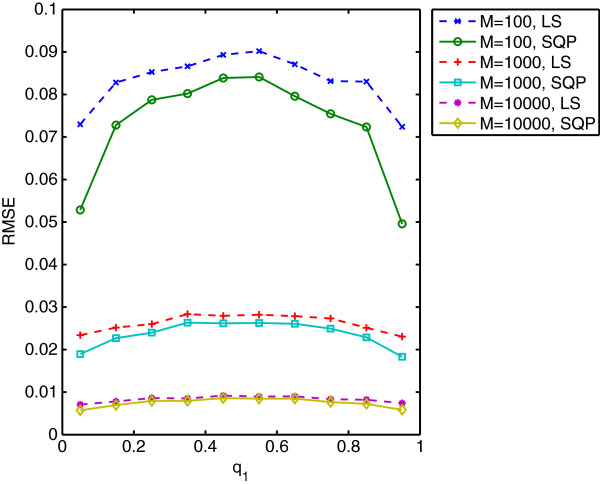
**Precision of best-case scenario for estimating Q. **Solid and dashed lines correspond to *Admixture*’s Sequential Quadratic Programming optimization and the least-squares approximation, respectively.

Figure 2 illustrates the root mean squared error in estimating **p** given the true value of **Q**. Both algorithms present the same pattern of performance as a function of **p** = [*p*_1_, *p*_2_]. Values of **p** near 0.5 present the most difficult scenarios. Positively correlated values (*e.g.*, *p*_1_ = *p*_2_) present slightly less error than negatively correlated values (*e.g.*, *p*_1_ = 1 – *p*_2_). Table 2 summarizes the performance over all values of **p** for varying *N* and *α*. In all cases, fmincon performs slightly better than least-squares and both algorithms approach zero error as *N* increases. We repeat this analysis for known values for **P** and estimate **q** using the two approaches. Figure 3 illustrates the difference in performance for the two algorithms as we vary *q*_1_ between 0.05 and 0.95 with *q*_2_ =1 – *q*_1_. Again, fmincon performs slightly better in all cases but both approach zero as *M* increases. In the next section we show that the additional error introduced by the least-squares approximation to the objective function remains small relative to the error introduced by the characteristics of the genotype data.

**Table 2:** **Root mean squared error in P for known Q and *K***= 2

**RMSE (%)**	***N= 100***			***N= 1000***			***N= 10000***		
	***α=0.1***	***α=1.0***	***α=2.0***	***α*=0.1**	***α*=1.0**	***α*=2.0**	***α*=0.1**	***α*=1.0**	***α*=2.0**
SQP	4.35	6.03	7.41	1.37	1.90	2.37	0.43	0.60	0.75
LS	4.37	6.16	7.68	1.38	1.93	2.40	0.44	0.61	0.76

### Simulated experiments to compare least-squares to *Admixture* and *FRAPPE*

**Figure 4:**
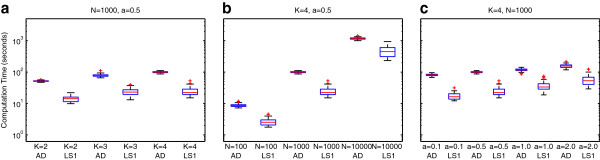
**Computational timing comparison. **Box plots show the median (red line) and inter-quartile range (blue box) for computation time on a logarithmic scale using (**a**) N=1000, α=0.5, and varying K; (**b**) K=4, α=0.5, and varying N; and (**c**) K=4, N=1000, and varying α.

In the previous sections, we consider the best-case scenario where the true value of **P** or **Q** is known. In a realistic scenario, the algorithms must estimate both **P** and **Q** from only the genotype information. Table 3 summarizes the results of a four-way analysis of variance with 2-way interactions among experimental factors. By far the factor with the most impact on performance is the number of individuals, *N*. The degree of admixture, *α*, and the number of populations, *K*, accounts for the second and third most variation, respectively. These three factors and two-way interactions between them account for the vast majority of variation. In particular, the choice of algorithm accounts for less than about 1% of the variation in estimation performance. That is, when estimating population structure from genotype data, the number of samples, the number of populations, and the degree of admixture play a much more important role than the choice between least-squares, *Admixture*, and *FRAPPE* and least-squares. However, as shown in Figure 4, when considering the computation time required by the algorithm, the choice of algorithm contributes about 40% of the variation including interactions. Therefore, for the range of population inference problems described in this study, the choice of algorithm plays a very small role in the estimation of **P** and **Q** but a larger role in computation time.

**Table 3:** Sources of variation in root mean squared error

**ANOVA**	**Error variance for P**		**Error variance for Q**		**Time variance**	
**Factors and interactions**	**Sum squared error (×10**^**-2**^**)**	**Percent**	**Sum squared error (×10**^**-4**^**)**	**Percent**	**Sum squared error (×10**^**4**^**)**	**Percent**
*K*	59.0	8.2	44.0	3.9	58.7	3.2
*N*	519.6	72.4	376.2	33.0	585.5	32.2
*Α*	63.1	8.8	341.1	29.9	33.2	1.8
Algorithm	0.1	0.0	1.7	0.1	266.3	14.6
*K* × *N*	32.1	4.5	32.6	2.9	98.2	5.4
*K* × *α*	9.0	1.3	8.2	0.7	4.4	0.2
*K* × Algorithm	0.0	0.0	0.4	0.0	55.1	3.0
*N* × *α*	29.1	4.1	282.6	24.8	58.8	3.2
*N* × Algorithm	0.0	0.0	2.1	0.2	445.6	24.5
*Α* × Algorithm	0.2	0.0	8.4	0.7	10.5	0.6
Error	5.7	0.8	43.2	3.8	204.4	11.2
Total	717.9	100.0	1140.4	100.0	1820.4	100.0

Further exploration reveals that the preferred algorithm depends on *K, N*, and *α*. Table 4 lists the root mean squared error for the estimation of **Q** for all combinations of parameters across *n* = 50 trials. Out of the 36 scenarios, *Admixture*, least-squares, and *FRAPPE* perform significantly better than their peers 13, six, and zero times, respectively; they perform insignificantly worse than the best algorithm 30, 17, and 10 times, respectively. The least-squares algorithm appears to perform well on the more difficult problems with combinations of large *K*, small *N*, or large *α*. Table 5 lists the root mean squared error for estimating **P**. For *N* = 100, the algorithms do not perform significantly differently. For *N* = 10000, all algorithms perform with less than 2.5% root mean squared error (RMSE). In all, *Admixture* performs significantly better than its peers 11 times out of 36. However, *Admixture* never performs significantly worse than its peers. Least-squares and *FRAPPE* perform insignificantly worse than *Admixture* 17 and 20 times out of 36, respectively. Table 6 summarizes the timing results. Least square converges significantly faster 34 out of 36 times with an insignificant difference for the remaining two scenarios. *FRAPPE* converges significantly slower in all scenarios. With two exceptions, the least-squares algorithm provides a 1.5- to 5-times speedup.

**Table 4:** Root mean squared error for Q

***K***	***N***	**α**	**AD**	**LS**	**FRAPPE**	**Significance**	**LS*α***
2	100	0.10	0.48	0.72	0.52	AD = FR < LS	0.64
2	100	0.50	1.12	1.13	1.03	FR = AD = LS	1.18
2	100	1.00	2.22	2.22	2.29	AD = LS = FR	2.22
2	100	2.00	4.13	4.11	4.50	LS = AD = FR	3.84
2	1000	0.10	0.57	0.97	0.63	AD < FR < LS	0.74
2	1000	0.50	0.69	0.74	0.71	AD < FR < LS	0.74
2	1000	1.00	0.86	0.91	1.00	AD < LS < FR	0.91
2	1000	2.00	1.58	1.65	2.33	AD = LS < FR	0.93
2	10000	0.10	0.59	1.03	0.61	AD < FR < LS	0.76
2	10000	0.50	0.70	0.81	0.72	AD < FR < LS	0.73
2	10000	1.00	0.74	0.77	0.79	AD < LS < FR	0.77
2	10000	2.00	0.89	0.97	1.32	AD < LS < FR	0.96
3	100	0.10	0.62	0.74	0.63	AD = FR < LS	0.66
3	100	0.50	2.01	1.81	2.00	LS < FR = AD	1.91
3	100	1.00	3.49	3.23	3.60	LS < AD = FR	3.23
3	100	2.00	5.77	5.39	5.89	LS < AD = FR	5.00
3	1000	0.10	0.68	1.15	0.73	AD < FR < LS	0.76
3	1000	0.50	0.85	0.88	0.89	AD < LS = FR	0.93
3	1000	1.00	1.18	1.17	1.35	LS = AD < FR	1.17
3	1000	2.00	1.94	1.92	2.49	LS = AD < FR	1.20
3	10000	0.10	0.74	1.26	0.76	AD < FR < LS	0.79
3	10000	0.50	0.87	0.97	0.87	AD = FR < LS	0.87
3	10000	1.00	0.89	0.92	0.95	AD < LS < FR	0.92
3	10000	2.00	1.07	1.09	1.49	AD < LS < FR	1.09
4	100	0.10	0.79	0.76	0.80	LS = AD = FR	0.77
4	100	0.50	2.81	2.40	2.85	LS < AD = FR	2.56
4	100	1.00	4.43	4.01	4.55	LS < AD = FR	4.01
4	100	2.00	6.63	6.13	6.81	LS < AD = FR	5.65
4	1000	0.10	0.73	1.17	0.74	AD = FR < LS	0.72
4	1000	0.50	0.95	0.95	1.00	LS = AD < FR	1.07
4	1000	1.00	1.34	1.32	1.47	LS = AD < FR	1.32
4	1000	2.00	2.09	2.06	2.50	LS = AD < FR	1.32
4	10000	0.10	0.84	1.33	0.84	AD = FR < LS	0.74
4	10000	0.50	0.96	1.03	0.96	AD = FR < LS	0.95
4	10000	1.00	0.97	0.99	1.03	AD < LS < FR	0.99
4	10000	2.00	1.14	1.15	1.51	AD = LS < FR	1.15

‘AD’ = *Admixture* with *ε* = *MN*×10^-4^, ‘LS1’ = Least-squares with *ε* = *MN*×10^-4^ and *α* = 1, ‘FR’ = *FRAPPE* with *ε* = 1. Bold values indicate significantly less error than those without bold. ‘<’ indicates significantly less at 4.6e-4 level, and ‘=’ indicates insignificant difference. ‘LS*α*’ = Least-squares with correct *α* provided only for reference.

**Table 5:** **Root mean squared error for** P

***K***	***N***	**α**	**AD**	**LS**	**FRAPPE**	**Significance**	**LS*α***
2	100	0.10	4.33	4.37	4.33	AD = FR = LS	4.36
2	100	0.50	5.13	5.17	5.14	AD = FR = LS	5.17
2	100	1.00	5.99	6.03	5.99	AD = FR = LS	6.03
2	100	2.00	7.24	7.28	7.29	AD = LS = FR	7.25
2	1000	0.10	1.37	1.42	1.38	AD < FR < LS	1.39
2	1000	0.50	1.62	1.65	1.63	AD = FR < LS	1.65
2	1000	1.00	1.90	1.93	1.92	AD < FR = LS	1.93
2	1000	2.00	2.52	2.58	2.82	AD = LS < FR	2.38
2	10000	0.10	0.46	0.57	0.46	AD < FR < LS	0.48
2	10000	0.50	0.52	0.56	0.53	AD < FR < LS	0.52
2	10000	1.00	0.60	0.61	0.62	AD < LS < FR	0.61
2	10000	2.00	0.81	0.87	1.14	AD < LS < FR	0.92
3	100	0.10	5.58	5.64	5.58	AD = FR = LS	5.62
3	100	0.50	7.37	7.42	7.38	AD = FR = LS	7.42
3	100	1.00	9.05	9.06	9.06	AD = FR = LS	9.06
3	100	2.00	11.36	11.33	11.39	LS = AD = FR	11.30
3	1000	0.10	1.78	1.87	1.78	AD = FR < LS	1.80
3	1000	0.50	2.35	2.40	2.35	AD = FR < LS	2.39
3	1000	1.00	2.97	3.00	3.01	AD < LS = FR	3.00
3	1000	2.00	4.11	4.14	4.41	AD = LS < FR	3.89
3	10000	0.10	0.61	0.82	0.62	AD < FR < LS	0.61
3	10000	0.50	0.78	0.84	0.78	AD = FR < LS	0.76
3	10000	1.00	0.93	0.95	0.98	AD < LS < FR	0.95
3	10000	2.00	1.35	1.36	1.82	AD = LS < FR	1.49
4	100	0.10	6.83	6.90	6.84	AD = FR = LS	6.87
4	100	0.50	9.61	9.63	9.62	AD = FR = LS	9.62
4	100	1.00	11.90	11.89	11.92	LS = AD = FR	11.89
4	100	2.00	14.94	14.89	15.01	LS = AD = FR	14.89
4	1000	0.10	2.16	2.28	2.16	AD = FR < LS	2.17
4	1000	0.50	3.10	3.15	3.11	AD = FR < LS	3.15
4	1000	1.00	4.04	4.06	4.08	AD < LS = FR	4.06
4	1000	2.00	5.61	5.62	5.88	AD = LS < FR	5.36
4	10000	0.10	0.76	1.02	0.77	AD = FR < LS	0.71
4	10000	0.50	1.04	1.11	1.04	AD = FR < LS	1.01
4	10000	1.00	1.28	1.30	1.33	AD < LS < FR	1.30
4	10000	2.00	1.87	1.87	2.36	AD = LS < FR	2.06

‘AD’ = *Admixture* with *ε* = *MN*×10^-4^, ‘LS1’ = Least-squares with *ε* = *MN*×10^-4^ and *α* = 1, ‘FR’ = *FRAPPE* with *ε* = 1. Bold values indicate significantly less error than those without bold. ‘<’ indicates significantly less at 4.6e-4 level, and ‘=’ indicates insignificant difference. ‘LS*α*’ = Least-squares with correct *α* provided only for reference.

**Table 6:** Computation time

***K***	***N***	**Α**	**AD**	**LS**	**FRAPPE**	**Significance**	**LS*α***
2	100	0.10	4.71	1.00	9.97	LS < AD < FR	0.77
2	100	0.50	4.69	1.16	8.22	LS < AD < FR	1.12
2	100	1.00	5.46	1.78	8.31	LS < AD < FR	1.77
2	100	2.00	6.25	2.37	10.40	LS < AD < FR	2.55
2	1000	0.10	43.37	11.87	136.88	LS < AD < FR	8.06
2	1000	0.50	51.70	13.98	112.41	LS < AD < FR	12.34
2	1000	1.00	62.00	24.43	118.90	LS < AD < FR	24.03
2	1000	2.00	83.07	51.33	195.43	LS < AD < FR	48.43
2	10000	0.10	447.68	142.14	1963.83	LS < AD < FR	93.61
2	10000	0.50	570.12	209.39	1908.72	LS < AD < FR	157.44
2	10000	1.00	687.88	352.24	2242.18	LS < AD < FR	349.51
2	10000	2.00	1037.45	796.83	3762.70	LS < AD < FR	406.63
3	100	0.10	6.10	1.84	15.29	LS < AD < FR	1.48
3	100	0.50	6.42	2.05	15.75	LS < AD < FR	1.90
3	100	1.00	7.19	2.71	16.78	LS < AD < FR	2.74
3	100	2.00	9.00	4.01	19.80	LS < AD < FR	4.24
3	1000	0.10	69.41	18.32	223.32	LS < AD < FR	12.53
3	1000	0.50	78.73	24.10	264.85	LS < AD < FR	21.42
3	1000	1.00	96.89	38.06	305.50	LS < AD < FR	36.63
3	1000	2.00	121.45	60.79	355.51	LS < AD < FR	55.54
3	10000	0.10	791.36	155.56	3256.83	LS < AD < FR	121.19
3	10000	0.50	883.99	301.52	4251.68	LS < AD < FR	264.77
3	10000	1.00	1175.25	617.80	5111.92	LS < AD < FR	578.42
3	10000	2.00	1506.20	1404.27	7052.33	LS < AD < FR	901.56
4	100	0.10	8.06	2.45	23.93	LS < AD < FR	2.00
4	100	0.50	8.78	2.66	26.56	LS < AD < FR	2.72
4	100	1.00	10.03	3.70	30.89	LS < AD < FR	3.43
4	100	2.00	12.94	5.00	37.26	LS < AD < FR	4.86
4	1000	0.10	81.72	17.32	386.11	LS < AD < FR	13.45
4	1000	0.50	99.92	24.37	433.17	LS < AD < FR	22.68
4	1000	1.00	117.71	36.94	508.49	LS < AD < FR	36.01
4	1000	2.00	156.39	58.02	564.57	LS < AD < FR	57.62
4	10000	0.10	879.95	229.06	5798.15	LS < AD < FR	176.27
4	10000	0.50	1170.97	480.99	7051.69	LS < AD < FR	505.45
4	10000	1.00	1555.90	1017.41	8108.08	LS < AD < FR	1051.81
4	10000	2.00	2202.08	2538.54	10445.75	AD = LS < FR	1308.79

‘AD’ = *Admixture* with *ε* = *MN*×10^-4^, ‘LS1’ = Least-squares with *ε* = *MN*×10^-4^ and *α* = 1, ‘FR’ = *FRAPPE* with *ε* = 1. Bold values indicate significantly less error than those without bold. ‘<’ indicates significantly less at 4.6e-4 level, and ‘=’ indicates insignificant difference. ‘LS*α*’ = Least-squares with correct *α* provided only for reference.

### Comparison on admixtures derived from the HapMap3 dataset

Tables 7 and 8 lists the performance and computation time for the least-squares approach and *Admixture* using a convergence threshold of *ε* = 1.0e-4 and *ε* = 1.4e-3, respectively. Each marker in the illustrations represents one individual. A short black line emanating from each marker indicates the offset from the original (correct) position. For all simulations, the least-squares algorithms perform within 0.1% of *Admixture* for estimating the true population allele frequencies in **P**. For well-mixed populations in Simulation 1 and 2, the least-squares algorithms perform comparably well or even better than *Admixture*. However, for less admixed data in Simulations 3 – 6, *Admixture* provides better estimates of the true population proportions depicted in the scatter plots. In all cases, the least-squares algorithms perform within 1.5% of *Admixture* and between about 2- and 3-times faster than *Admixture*.

**Table 7:** Simulation experiments (1–3) using realistic population allele frequencies from the HapMap phase 3 project

	**Simulation 1 q ~ Dir(1,1,1)**			**Simulation 2 q ~ Dir(.5,.5,.5)**			**Simulation 3 q ~ Dir(.1,.1,.1)**		
Original									
Admixture									
Least-squares (*α*=1)									
Least-squares with *α*									
	**RMSE (%) ± Std. Dev.**		**Time (s.) ± Std. Dev.**	**RMSE (%) ± Std. Dev.**		**Time (s.) ± Std. Dev.**	**RMSE (%) ± Std. Dev.**		**Time (s.) ± Std. Dev.**
	**P**	**Q**		**P**	**Q**		**P**	**Q**	
AD (*ε*=1e-4)	2.50 ± 0.04	2.19 ± 0.11	105 ± 13	1.99 ± 0.02	1.44 ± 0.04	88 ± 9	1.54 ± 0.01	0.76 ± 0.02	86 ± 7
AD (*ε*=1.4e-3)	2.50 ± 0.04	2.19 ± 0.11	98 ± 13	1.99 ± 0.02	1.44 ± 0.04	87 ± 11	1.54 ± 0.01	0.76 ± 0.02	83 ± 9
LS1 (*ε*=1.4e-3)	2.51 ± 0.03	1.85 ± 0.07	51 ± 6	2.04 ± 0.02	1.43 ± 0.04	37 ± 8	1.63 ± 0.01	1.75 ± 0.05	27 ± 5
LS*α *(*ε*=1.4e-3)	2.51 ± 0.03	1.85 ± 0.07	54 ± 8	2.03 ± 0.02	1.53 ± 0.04	28 ± 4	1.57 ± 0.01	1.08 ± 0.02	15 ± 4

**Table 8:** Simulation experiments (4–6) using realistic population allele frequencies from the HapMap phase 3 project

	**Simulation 4 q ~ Dir(.2,.2,.05)**			**Simulation 5 q ~ Dir(.2,.2,.5)**			**Simulation 6 q ~ Dir(.05,.05,.01)**		
Original									
Admixture									
Least-squares (*α*=1)									
Least-squares with *α*									
	**RMSE (%) Std. Dev.**		**Time (s.) ± Std. Dev.**	**RMSE (%) ± Std. Dev.**		**Time (s.) ± Std. Dev.**	**RMSE (%) ± Std. Dev.**		**Time (s.) ± Std. Dev.**
	**P**	**Q**		**P**	**Q**		**P**	**Q**	
AD (*ε*=1e-4)	2.01 ± 0.05	0.87 ± 0.02	94 ± 12	1.98 ± 0.03	1.16 ± 0.03	93 ± 17	1.96 ± 0.07	0.53 ± 0.02	91 ± 9
AD (*ε*=1.4e-3)	2.01 ± 0.05	0.87 ± 0.02	82 ± 5	1.98 ± 0.03	1.16 ± 0.03	86 ± 13	1.96 ± 0.07	0.53 ± 0.02	82 ± 7
LS1 (*ε*=1.4e-3)	2.09 ± 0.05	1.70 ± 0.05	31 ± 7	2.06 ± 0.03	1.60 ± 0.04	34 ± 5	2.04 ± 0.07	2.00 ± 0.04	27 ± 7
LS*α *(*ε*=1.4e-3)	2.05 ± 0.05	1.17 ± 0.03	17 ± 3	2.02 ± 0.04	1.34 ± 0.04	24 ± 4	1.99 ± 0.07	1.09 ± 0.03	14 ± 3

The apparent advantage of *Admixture* involves individuals on the periphery of the unit simplex defining the space of **Q**. In Table 7, this corresponds to individuals on the boundary of the right triangle defined by the *x*-axis, *y*-axis, and *y* = 1 – *x* diagonal line. For Simulation 1, the original **Q** contains very few individuals on the boundary, *Admixture* estimates far more on the boundary, and the least-squares was closer to the ground truth. For Simulation 2 – 6, the ground truth contains more individuals on the boundary, *Admixture* correctly estimates these boundary points but the least-squares algorithms predict fewer points on the boundary. Simulation 6 provides the most obvious example where *Admixture* estimates individuals exactly on the boundary and least-squares contains a jumble of individuals near but not exactly on the line.

### Real dataset from the HapMap phase 3 project

**Figure 5:**
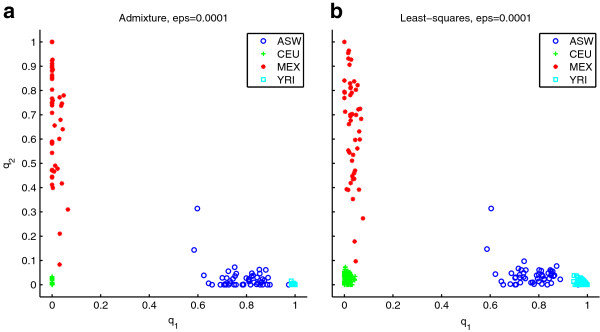
**Comparison on HapMap Phase 3 dataset. **Inferred population membership proportions using (**a**) *Admixture *and (**b**) least-squares with *α*=1. Each point represents a different individual among the four populations: ASW, CEU, MEX, and YRI. The axes represent the proportion of each individual’s genome originating from each inferred population. The proportion belonging to the third inferred population is given by q_3 _= 1 – q_1 _– q_2_.

Over 20 repeated trials, *Admixture* converged in an average of 42.1 seconds with standard deviation of 9.1 seconds, and the least-squares approach converged in 33.6 seconds with a standard deviation of 9.8 seconds. Figure 5 illustrates the inferred population proportions for one run. The relative placement of individuals from each known population is qualitatively similar. The two methods differ at extreme points such as those values of *q*_1_, *q*_2_, or 1 – *q*_1_ – *q*_2_ that are near zero. The *Admixture* solution has more individuals on the boundary and the least-squares approach has fewer. Although we cannot estimate the error of these estimates because the real world data has no ground truth, we can compare their results quantitatively. The *Admixture* and the least-squares solution differed by an average of 1.2% root mean squared difference across the 20 trials. We estimate *α* = 0.12 from the *Admixture* solution’s total variance using Equation 31. This roughly corresponds to the simulated experiment with three populations, 100 samples, and a degree of admixture of 0.1. In that case, *Admixture* and least-squares exhibited a very small root mean squared error of 0.62% and 0.74%, respectively (Table 4).

## Discussion

This work contributes to the population inference literature by providing a novel simplification of the binomial likelihood model that improves the computational efficiency of discrete admixture inference. This approximation results in an inference algorithm based on minimizing the squared distance between the genotype matrix **G** and twice the product of the population allele frequencies and individual admixture proportions: 2**PQ**. This Euclidean distance-based interpretation aligns with previous results employing multivariate statistics. For example, researchers have found success using principal component analysis to reveal and remove stratification [2-4] or even to reveal clusters of individuals in subpopulations [5-7]. Recently, McVean [5] proposed a genealogical interpretation of principal component analysis and uses it to reveal information about migration, geographic isolation, and admixture. In particular, given two populations, individuals cluster along the first principal component. Admixture proportion is the fractional distance between the two population centers. However, these cluster centers must known or inferred in order to estimate ancestral population allele frequencies. The least-squares approach infers these estimates efficiently and directly.

Typically, discrete admixture models employ a binomial likelihood function rather than a Euclidean distance-based one. Pritchard et al. detail one such model and use a slow sampling based approach to infer the admixed ancestral populations for individuals in a sample [9]. Recognizing the performance advantage of maximizing the likelihood rather than sampling the posterior, Tang et al. proposed an expectation maximization algorithm and Alexander et al. [13] proposed a sequential quadratic programming (SQP) approach using the same likelihood function [9]. We take this approach a step further by simplifying the model proposed by Pritchard et al. to introduce a least-squares criterion. By justifying the least-squares simplification, we connect the fast and practical multivariate statistical approaches to the theoretically grounded binomial likelihood model. We validate our approach on a variety of simulated and real datasets.

First, we show that if the true value of **P** (or **Q**) is known, the expected value of the least squares solution for **Q** (or **P**) across all possible genotype matrices is equal to the true value, and the variance of this estimate decreases with *M* (or *N*). In this best-case scenario, we show that SQP provides a slightly better estimate than the least-squares solution for a variety of problem sizes and difficulty. For more common scenarios where the algorithms must estimate **P** and **Q** using only the genotype information in **G**, we show that for particularly difficult problems with small *N*, large *K*, or large *α*, the least-squares approach often performs better than its peers. For about one-third of the parameter sets, *Admixture* performs significantly better than least-squares and *FRAPPE* but all algorithms approach zero error as *N* becomes very large. In addition, the error introduced by the choice of algorithms was relatively small compared to other characteristics of the experiment such as sample size, number of populations, and the degree of admixture in the sample. That is, improving accuracy has more to do with improving the dataset than with selecting the algorithm, suggesting that algorithm selection may depend on other criteria such as its speed. In nearly all cases, the least-squares method computes its solution faster, typically a 1.5- to 5-times faster. At the current problem size involving about 10000 loci, this speed improvement may justify the use of least-squares algorithms. For a single point estimate, researchers may prefer a slightly more accurate algorithm at the cost of seconds or minutes. For researchers testing several values of *K* and *α* and using multiple runs to gauge the fitness of each parameter set, or those estimating standard errors [13], the speed improvement could be the difference between hours and days of computation. As the number of loci increase to hundreds of thousands or even millions, speed may be more important. The least-squares approach offers an alternative simpler and faster algorithm for population inference that provides qualitatively similar results.

The key speed advantage of the least-squares approach comes from a single nonnegative least-squares update that minimizes a quadratic criterion for **P** and then for **Q** per iteration. *Admixture*, on the other hand, minimizes several quadratic criteria sequentially as it fits the true binomial model. Although the least-squares algorithm completes each update in less time and is guaranteed to converge to a local minimum or straddle point, predicting the number of iterations to convergence presents a challenge. We provide empirical timing results and note that selecting a suitable stopping criterion for these iterative methods can change the timing and accuracy results. For comparison, we use the same stopping criterion with published thresholds for *Admixture* and *FRAPPE*[13], and a threshold of *MN*×10^-10^ for least-squares.

This work is motivated in part by the desire to analyze larger genotype datasets. In this paper, we focus on the computational challenges of analyzing very large numbers of markers and individuals. However, linkage disequilibrium introduces correlations between loci that cannot be avoided in very large datasets. Large datasets can be pruned to diminish the correlation between loci. For example, Alexander et al. prune the HapMap phase 3 dataset from millions of SNPs down to around 10000 to avoid correlations. In this study, we assume linkage equilibrium and therefore uncorrelated markers and limit our analysis to datasets less than about 10000 SNPs. Incorporating linkage disequilibrium in gradient-based optimizations of the binomial likelihood model remains an open problem.

Estimating the number of populations *K* from the admixed samples continues to pose a difficult challenge for clustering algorithms in general and population inference in particular. In practice, experiments can be designed to include individual samples that are expected to be distributed close to their ancestors. For example, Tang et al. [11] suggested using domain knowledge to collect an appropriate number of pseudo-ancestors that reveal allele frequencies of the ancestral populations. The number of groups considered provides a convenient starting point for *K*. Lacking domain knowledge, computational approaches can be used to try multiple reasonable values for *K* and evaluating their fitness. For example, Pritchard et al. [9] estimated the posterior distribution of *K* and select the most probable *K*. Another approach is to evaluate the consistency of inference for different values of *K*. If the same value of *K* leads to very different inferences of **P** and **Q** from different random starting points, the inference can be considered inconsistent. Brunet et al. [18] proposed this method of model selection called consensus clustering.

For realistic population allele frequencies, **P**, from the HapMap Phase 3 dataset and very little admixture in **Q**, *Admixture* provides better estimates of **Q**. The key advantage of *Admixture* appears to be for individuals containing nearly zero contribution from one or more inferred populations, whereas the least-squares approach performs better when the individuals are well-mixed. Visually, both approaches reveal population structure. Using the two approaches to infer three ancestral populations from four HapMap Phase 3 sampling populations reveals qualitatively similar results.

We believe the computational advantage of the least-squares approach along with its good estimation performance warrants further research especially for very large datasets. For example, we plan to adapt and apply the least-squares approach to datasets utilizing microsatellite data rather than SNPs and consider the case of more than two alleles per locus. Researchers have incorporated geospatial information into sampling-based [19] and PCA-based [8] approaches. Multiple other extensions to sampling-based or PCA-based algorithms have yet to be incorporated into faster gradient-based approaches.

## Conclusion

This paper explores the utility of a least-squares approach for the inference of population structure in genotype datasets. Whereas previous Euclidean distance-based approaches received little theoretical justification, we show that a least-squares approach is the result of a first-order approximation of the negative log-likelihood function for the binomial generative model. In addition, we show that the error in this approximation approaches zero as the number of samples (individuals and loci) increases. We compare our algorithm to state-of-the-art algorithms, *Admixture* and *FRAPPE*, for optimizing the binomial likelihood model, and show that our approach requires less time and performs comparably well. We provide both quantitative and visual comparisons that illustrate the advantage of *Admixture* at estimating individuals with little admixture, and show that our approach infers qualitatively similar results. Finally, we incorporate a degree of admixture parameter that improves estimates for known levels of admixture without requiring additional parameter tuning as is the case for *Admixture*.

## Methods

The algorithms we discuss accept the number of populations, *K*, and an *M* × *N* genotype matrix, **G** as input:

(3) $$G=\left[\begin{array}{l} g_{11}\;g_{12}\;\cdots \;g_{1N}\\ g_{21}\;g_{22}\;\cdots \;g_{2N}\\ \vdots \;\vdots \;\ddots \;\vdots \\ g_{M1}\;g_{M2}\;\cdots \;g_{\text{MN}} \end{array}\;\right]$$

where *g*_*li*_ ∈ {0,1,2} representing the number of copies of the reference allele at the *l*th locus for the *i*th individual, *M* is the number of markers (loci), and *N* is the number of individuals. Given the genotype matrix, **G**, the algorithms attempt to infer the population allele frequencies and the individual admixture proportions. The matrix **P** contains the population allele frequencies:

(4) $$P=\left[\begin{array}{l} p_{11}\;p_{12}\;\cdots \;p_{1K}\\ p_{21}\;p_{22}\;\cdots \;p_{2K}\\ \;\vdots \;\vdots \;\ddots \;\vdots \\ p_{M1}\;p_{M2}\;\cdots \;p_{\text{MK}} \end{array}\;\right]$$

where 0 ≤ *p*_*lk*_ ≤ 1 representing the fraction of reference alleles out of all alleles at the *l*th locus in the *k*th population. The matrix **Q** contains the individual admixture proportions:

(5) $$Q=\left[\begin{array}{l} q_{11}\;q_{12}\;\cdots \;q_{1N}\\ q_{21}\;q_{22}\;\cdots \;q_{2N}\\ \;\vdots \;\vdots \;\ddots \;\vdots \\ q_{K1}\;q_{K2}\;\cdots \;q_{\text{KN}} \end{array}\;\right]$$

where 0 ≤ *q*_*ik*_ ≤ 1 represents the fraction of the *i*th individual’s genome originating from the *k*th population and for all *i*, ∑_*k*_*q*_*ki*_ = 1. Table 1 summarizes the matrix notation we use.

### Likelihood function

Alexander et al. model the genotype (*i.e.,* the number of reference alleles at a particular locus) as the result of two draws from a binomial distribution [13]. In the generative model, each allele copy for one individual at one locus has an equal chance, *m*_*li*_, of receiving the reference allele:

(6) $$m_{\text{li}}=\Sigma_{k=1}^{K}p_{1k}q_{k1}$$

The log-likelihood of the parameters **P** and **Q** from the original *Structure* binomial model and ignoring an additive constant is the following [13]:

(7) $$L\left(M\right)=\overset{M}{\underset{l=1}{\Sigma }}\overset{N}{\underset{i=1}{\Sigma }}g_{\text{li}}1n\left[m_{\text{li}}\right]+\left(2-g_{\text{li}}\right)1n\left[1-m_{\text{li}}\right]$$

To see the effect on gradient-based optimization, we also present the derivative of the likelihood with respect to a particular *m*_*li*_:

(8) $$\frac{\partial }{\partial m_{\text{li}}}L\left(M\right)=\frac{g_{\text{li}}-2m_{\text{li}}}{m_{\text{li}}\left(1-m_{\text{li}}\right)}\approx 4\left(g_{\text{li}}-2m_{\text{li}}\right)$$

**Figure 6:**
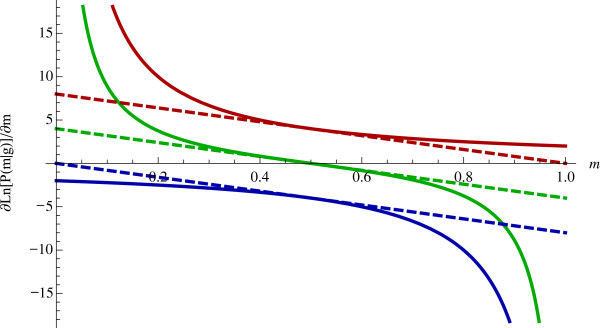
**First-order approximation for slope of log-likelihood of *m*. **Solid and dashed lines correspond to the true and approximated slope, respectively. The red, green, and blue lines correspond to *g *= 0, *g *= 1, and *g *= 2, respectively.

In order to achieve a least-squares criterion, we must approximate this derivative with a line. Figure 6 plots this derivative with respect to *m*_*li*_ for the three possible values of *g*_*li*_ (0, 1, or 2). To avoid biasing the approximation to high or low values of *m*_*li*_, we approximate the derivative with its first-order Taylor approximation in the neighborhood of *m*_*li*_ = 1/2. More complex optimizations might update the neighborhood of the Taylor approximation during the optimization. In the interest of simplicity, we select one neighborhood for all iterations, genotypes, individuals, and loci. The following least-squares objective function has the approximated derivative in the above equation:

(9) $$-L\left(M\right)\approx \overset{L}{\underset{l=1}{\Sigma }}\overset{N}{\underset{i=1}{\Sigma }}{\left(2m_{\text{li}}-g_{\text{li}}\right)}^{2}={\left\|2M-G\right\|}_{2}^{2}$$

The right-hand-side of Equation 9 provides the least-squares criterion. Figure 6 shows the deviation between the linear approximation and the true slope. Values match closely for 0.35 ≤ *m*_*li*_ ≤ 0.65 but as *m*_*li*_ approaches zero or one the true slope diverges for two of the three genotypes. Therefore, we have the following least-squares optimization problem:

(10) $$\underset{P,Q}{\text{arg}\;\text{min}}{\left\|2\text{PQ}-G\right\|}_{2}^{2},\text{such}\;\text{that}\{\begin{array}{l} 0\leq P\leq 1\\ \;Q\geq 0\\ \Sigma_{k=1}^{K}q_{\text{ki}}=1 \end{array}$$

### Bounded error for the least-squares approach

We justify the least-squares approach by showing that the expected value across all genotypes is equal to the true value in the binomial likelihood model, and that the covariance approaches zero as the size of the data increases. In order to analyze the least squares performance across all possible genotype matrices, we consider the generative model for **G**. Given the true ancestral population allele frequencies, **P**, and the proportion of each individual’s alleles originating from each population, **Q**, the genotype at locus *l* for individual *i* is a binomial random variable, *g*_*li*_:

(11) $$\begin{array}{l} g_{\text{li}}∼\text{Binomial}(2,m_{\text{li}})\\ m_{\text{li}}=\Sigma_{k=1}^{K}p_{1k}q_{\text{ki}} \end{array}$$

If **M** was directly observable, we could solve for **P** or **Q** given the other using **P** = **MQ**^**#**^ or **Q** = **P**^**#**^**M**, where # is the Moore-Penrose pseudo-inverse. However, we only observe the elements of **G** which is only partially informative of **M**. First we consider the uncertainty in estimating **P**. Each *g*_*li*_ is an independent random variable with the following mean and bound on the variance:

(12) $$\begin{array}{l} E[g_{\text{li}}]=2m_{\text{li}}\\ \text{var}[g_{\text{li}}]\frac{1}{2} \end{array}$$

### Mean and total variance of the estimate of p

For ease of notation, we focus on one locus at index *l* in one row of **P**,

$$\hat{p}={\left[{\hat{p}}_{l1},{\hat{p}}_{12},\ldots ,{\hat{p}}_{1K}\right]}^{T}$$,

one row of **G**, *g* = [*g*_*l*1_, *g*_12_, …, *g*_1*N*_]^*T*^, and estimate the mean, covariance, and provide a bound on the total variance of its estimate:

(13) $$\begin{array}{l} \hat{p}=\frac{1}{2}Q^{T}gE[\hat{p}]=p\text{cov}[\hat{p}]\\ \;=\frac{1}{4}Q^{T}\text{cov}[g]Qtrace(\text{cov}[\hat{p}])\leq \frac{1}{8}trace\left({\left(QQ^{T}\right)}^{-1}\right) \end{array}$$

Intuitively, **QQ**^*T*^ scales linearly with *N* and we expect the bound on the trace to decrease linearly with *N*. If the columns, **q**, of **Q** are independent and identically distributed, **QQ**^*T*^ approaches *N*×*E*[**qq**^*T*^], resulting in a bound that decreases linearly with *N*:

(14) $$\text{trace}\left(\text{cov}\left[\hat{p}\right]\right)\leq \frac{1}{8N}\text{trace}\left({\left(E\left[qq^{T}\right]\right)}^{-1}\right)$$

To put this bound in more familiar terms we consider **q** drawn from a Dirichlet distribution with shape parameter *α*, resulting in the following:

(15) $$E\left[qq^{T}\right]=\frac{1}{4\alpha +2}\left[\begin{array}{l} \alpha +1\;\alpha \\ \alpha \;\alpha +1 \end{array}\;\right]$$

Asymptotically, **QQ**^*T*^ approaches *N*×*E*[**qq**^*T*^] and (**QQ**^*T*^)^-1^ approaches:

(16) $$\frac{2}{N}\left[\begin{array}{l} \alpha +1\;-\alpha \\ -\alpha \;\alpha +1 \end{array}\;\right]$$

resulting in the following asymptotic bound on the total variance:

(17) $$\text{trace}\left(\text{cov}\left[\hat{p}\right]\right)\leq \frac{1}{4N}{\left(\alpha +\right)}^{2}$$

### Mean and total variance of the estimate for q

The same analysis can be repeated for one individual at index *i* in one column of **Q**,

$$\hat{q}={\left[{\hat{q}}_{\text{li}},{\hat{q}}_{2i},\ldots ,{\hat{q}}_{\text{ki}}\right]}^{T}$$

and one column of **G**, *g* = [*g*_*li*_, *g*_2*i*_, …, *g*_*Li*_]^*T*^:

(18) $$\begin{array}{l} \hat{q}=\frac{1}{2}PgE[\hat{q}]=qcov[\hat{q}]\\ \;=\frac{1}{4}P\text{cov}[g]P^{T}\text{trace}(\text{cov}[\hat{q}])\leq \frac{1}{8}\text{trace}\left({\left(P^{T}P\right)}^{-1}\right) \end{array}$$

Intuitively, **P**^*T*^**P** increases linearly with *M*, and we expect the bound on the total variance to decrease linearly with *M*. Similarly, if the rows, **p**, of **P** are independent and identically distributed, **P**^*T*^**P** approaches *M*×*E*[**pp**^*T*^], resulting in an asymptotic bound that decreases linearly with *M*:

(19) $$\text{trace}\left(\text{cov}\left[\hat{q}\right]\right)\leq \frac{1}{8M}\text{trace}\left({\left(E\left[p^{T}p\right]\right)}^{-1}\right)$$

### Incorporating degree of admixture, *α*

Pritchard et al. [13] use a prior distribution to bias their solution toward those with a desired level of admixture. This prior on the columns of **Q** takes the form of a Dirichlet distribution:

(20) $$q∼D\left(\alpha ,\alpha ,\ldots ,\alpha \right)$$

Because all the shape parameters (*α*) are equal, this prior assumes that all ancestral populations are equally represented in the current sample. The log of this prior probability is the following ignoring an additive constant:

(21) $$\begin{array}{l} \text{In}\;P\left(q\right)=\left(\alpha -1\right)\overset{K}{\underset{k=1}{\Sigma }}\text{ln}\left[q_{k}\right],\;\text{where}\;q_{k}\\ \;=1-\overset{K-1}{\underset{k=1}{\Sigma }}q_{k} \end{array}$$

The derivative of the log prior with respect to *q* and its first-order approximation at the mean of *q*_*k*_ = 1/*K* is the following:

(22) $$\begin{array}{l} \frac{\partial }{\partial q_{k}}\text{ln}\;P\left(q\right)=-\frac{\left(\alpha -1\right)\left(q_{k}-q_{K}\right)}{q_{k}q_{K}}\\ \;\approx -2K^{2}\left(\alpha -1\right)\left(q_{k}-\frac{1}{K}\right) \end{array}$$

The following penalty function combines the columns of **Q** into a single negative log-likelihood function with the approximated derivative in the above equation:

(23) $$\begin{array}{l} -\text{ln}\;p\left(Q\right)\approx K^{2}\left(\alpha -1\right)\overset{N}{\underset{i=l}{\Sigma }}\overset{K}{\underset{k=1}{\Sigma }}{\left(q_{\text{ki}}-\frac{1}{K}\right)}^{2}\\ \;=K^{2}\left(\alpha -1\right){\left\|Q-\frac{1}{K}\right\|}_{2}^{2} \end{array}$$

**Figure 7:**
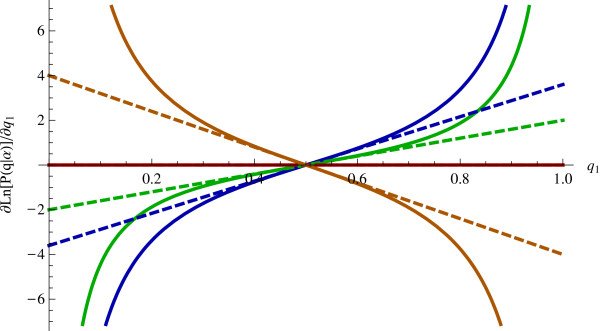
**First-order approximation for slope of log-likelihood of *q*. **Solid and dashed lines correspond to the true and approximated slope, respectively, for *K *= 2. The blue, green, red, and orange lines correspond to *α *= 0.1, *α *= 0.5, *α *= 1, and *α* = 2, respectively.

The right-hand-side of Equation 23 acts as a penalty term for the least-squares criterion in Equation 9. Figure 7 shows the difference between the real and approximated slope. For *q* near its mean of 1/*K*, the approximation fits closely but for extreme values of *q* the true slope diverges. Combining the terms in Equations 9 and 23 and including problem constraints, we have the following least-squares optimization problem:

(24) $$\begin{array}{l} \underset{P,Q}{\text{arg}\;\text{min}}{\left\|2\text{PQ}-G\right\|}_{2}^{2}\\ \;+K^{2}\left(\alpha -1\right){\left\|Q-\frac{1}{K}\right\|}_{2}^{2},\text{such}\;\text{that}\left\{\begin{array}{l} 0\leq P\leq 1\\ \;Q\geq 0\\ \Sigma_{k=1}^{K}q_{\text{ki}}=1 \end{array}\right) \end{array}$$

### Optimization algorithm

The non-convex optimization problem in Equation 10 can be approached as a two-block coordinate descent problem [15,20]. We initialize **Q** with nonnegative values such that each column sums to one. Then, we alternate between minimizing the criterion function with respect to **P** with fixed **Q**:

(25) $$\underset{0\leq P\leq }{\text{arg}\;\text{min}}{\left\|2\text{PQ}-G\right\|}_{2}^{2}$$

and then minimizing with respect to **Q** with fixed **P**:

(26) $$\underset{\underset{\;\Sigma_{k=1}^{K}q_{\text{ki}}=1}{Q\geq 0}}{\text{arg}\;\text{min}}{\left\|2\text{PQ}-G\right\|}_{2}^{2}+K^{2}\left(\alpha -1\right){\left\|Q-\frac{1}{K}\right\|}_{2}^{2}$$

This process is repeated until the change in the criterion function is less than *ε* at which point we consider the algorithm to have converged. The *Admixture* algorithm suggests a threshold of *ε* = 1e-4 but we have found that a larger threshold often suffices. Unless otherwise stated, we use a threshold that depends on the size of the problem: *ε* = *MN*×10^-10^, corresponding to 1e-4 when *M* = 10000 and *N* = 100.

### Least-squares solution for P

Van Benthem and Keenan [16] propose a fast nonnegatively constrained active/passive set algorithm that avoids redundant calculations for problems with multiple right-hand-sides. Without considering the constraints on **P**, Equation 25 can be classically solved using the pseudo-inverse of **Q**:

(27) $$\hat{P}=\frac{1}{2}GQ^{T}{\left(QQ^{T}\right)}^{-1}$$

However, some of the elements of **P** may be less than zero. In the active/passive set approach, if elements of **P** are negative, they are clamped at zero and added to the active set. The unconstrained solution is then applied to the remaining passive elements of **P**. If the solution happens to be nonnegative, the algorithm finishes. If not, negative elements are added to the active set and elements in the active set with a negative gradient (will decrease the criterion by increasing) are added back to the passive set. The process is repeated until the passive set is non-negative and the active set contains only elements with a positive gradient at zero. We extend the approach of Van Benthem and Keenan to include an upper bound at one. Therefore, we maintain two active sets: those clamped at zero and those clamped at one and update both after the unconstrained optimization of the passive set at each iteration. We provide Matlab source code that implements this algorithm on our website.

### Least-squares solution for Q

When solving for **Q** it is convenient to reformulate Equation 26 into simpler terms:

(28) argminQ≥0Σk=1Kqki=1P¯Q−G¯22P¯=2PKα−112IKG¯=Gα−1121KxN

The unconstrained solution for this equation is the following:

(29) $$\begin{array}{ll} \hat{Q} & ={\left(4P^{T}P+K^{2}\left(\alpha -1\right)I\right)}^{-1}\left(2P^{T}G+K\left(\alpha -1\right)\right)\\ & ={\left({\bar{P}}^{T}\bar{P}\right)}^{-1}{\bar{P}}^{T}\bar{G} \end{array}$$

When prior information is known about the sparseness, we use *α* in the equations above. When no prior information is known, we use *α* = 1 corresponding to the uninformative prior and resulting in the ordinary pseudo-inverse solution. In order to incorporate the sum-to-one constraint on the columns of **Q**, we employ the method of Lagrange multipliers using Equation 11 in the work of Settle and Drake substituting the identity matrix for the noise matrix, **N**[21]. For completeness, we include the solution below:

(30) $$\begin{array}{l} Q=aUj+(U-aUJU){\bar{P}}^{T}\bar{G}\\ U={\left({\bar{P}}^{T}\bar{P}\right)}^{-1}\\ a={\left(\overset{K}{\underset{i=1}{\Sigma }}\overset{K}{\underset{j=1}{\Sigma }}u_{\mathrm{ij}}\right)}^{-1}\\ j={\left[1,1,\ldots ,1\right]}^{T}\\ J=jj^{T} \end{array}$$

As before, some elements of **Q** may be negative. In that case, we utilize the active set method to clamp elements of **Q** at zero and update active and passive sets at each iteration until convergence as described above. We adapt the Matlab script by Van Benthem and Keenan so that the unconstrained solution uses Equation 30 instead of the standard pseudo-inverse and provide it on our website.

### Simulated experiments to compare the proposed approach to *Admixture* and *FRAPPE*

We generate simulated genotype data for a variety of problems using *M* = 10000 markers, and varying *N* between 100, 1000, and 10000; *K* between 2, 3, and 4; and *α* between 0.1, 0.5, 1, and 2, for a total of 36 parameter sets. For each combination of *N*, *K*, and *α*, we generate the ground truth **P** from a uniform distribution, and **Q** from a Dirichlet distribution parameterized by *α*. Then, we draw a random genotype for each individual using the binomial distribution in Equation 11. We estimate **P** and **Q** using only the genotype information and the true number of populations, *K*. We repeat the experiment 50 times drawing new, **P**, **Q**, and **G** matrices each time. Finally, we record the performance of *Admixture* using the published tight convergence threshold of *ε* = 1e-4[13] and a loose convergence threshold of *ε* = *MN*×10^-4^; the least-squares algorithm using an uninformative prior (*α* = 1) and *ε* = *MN*×10^-4^, and the *FRAPPE EM* algorithm using the published threshold of *ε* = 1. For reference, we also include the least-squares algorithm with informative prior (known *α*) with convergence threshold of *ε* = *MN*×10^-4^_._ In all experiments, *Admixture*’s performances with the two convergence thresholds were nearly identical and we only report the results for *ε* = *MN*×10^-4^, resulting in shorter computation times. We used a four-way analysis of variance (ANOVA) with a fixed effects model to reveal which factors (including algorithm) contribute more or less to the estimation error and computation time.

### Statistical significance of root mean squared error and computation time

For each combination of *K*, *N*, and *α*, we perform a Kruskal-Wallis test to determine if *Admixture*, Least-Squares, and *FRAPPE* perform significantly differently at a Bonferroni adjusted significance level of 0.05/(36 parameter sets) = 0.0014. If there is no significant difference, we consider their performances equal. If there is a significant difference, we perform pair-wise Mann–Whitney U-tests to determine significant differences between specific algorithms. We use a Bonferroni adjusted significance level of 0.05/(36 parameter sets)/(3 pair-wise comparisons) = 4.6e-4. The ‘Summary’ columns contain the order of performance among the algorithms such that every algorithm to the left of a ‘<’ symbol performs better than every algorithm to the right. An ‘=’ symbol indicates that the adjacent algorithms do not perform significantly differently.

### Comparison on admixtures derived from the HapMap3 dataset

In the original *Admixture* paper [13], the authors simulate admixed genotypes from population allele frequencies derived from the HapMap Phase 3 dataset [22]. We follow their example to compare the algorithms with more realistic population allele frequencies. Rather than drawing **P** from a uniform distribution, we estimate the population allele frequencies for unrelated individuals in the HapMap Phase 3 dataset using individuals from the following groups: Han Chinese in Beijing, China (CHB), Utah residents with ancestry from Northern and Western Europe (CEU), and Yoruba individuals in Ibadan, Nigeria (YRI) [22]. We use the same 13928 SNPs provided in the sample data on the *Admixture* webpage [23]. We randomly simulate 1000 admixed individuals: **q** ~ Dirichlet(*α*_*1*_, *α*_*2*_, *α*_*3*_). When the Dirichlet parameters are not equal, we use the degree of admixture, *α*, for LS*α* that results in the same total variance as the combination of *α*_*1*_, *α*_*2*_, and *α*_*3*_:

(31) $$\begin{array}{c} \alpha =\frac{K-1}{K^{2}v}-\frac{1}{K},\\ \text{where}\;\text{the}\;\text{total}\;\text{variance},\;v=\Sigma_{k=1}^{K}\frac{\alpha_{k}\left(\alpha_{0}-\alpha_{k}\right)}{\alpha_{0}^{2}\left(\alpha_{0}+1\right)},\\ \;\text{and}\;\alpha_{0}=\Sigma_{k=1}^{K}\alpha_{k} \end{array}$$

### Real dataset from the HapMap phase 3 project

In the original *Admixture* paper [13], the authors use *Admixture* to infer three hypothetical ancestral populations from four known populations in the HapMap Phase 3 dataset, including individuals with African ancestry in the American Southwest (ASW), individuals with Mexican ancestry in Los Angeles (MEX), and the same CEU CEU and YRI individuals from the previous example. We ran each algorithm 20 times on the dataset using a convergence threshold of *ε* = 1e-4, recording the convergence times for each trial.

## Competing interests

The authors declare that they have no competing interests.

## Authors’ contributions

RMP conceived of the least-squares approach to inferring population structure, designed the study, and drafted the document. MDW initiated the SNP data analysis project, acquired funding to sponsor this effort, and directed the project and publication. All authors read and approved the final manuscript.
